# Cases of Gunshot Injury Requiring Ligation of the Artery. (From Reports in Surgeon-General's Office.)

**Published:** 1864-11

**Authors:** H. L. Thomas


ART. IV.—*Cases of Gunshot Injury Requiring Ligation of the Artery.* (From Reports in Surgeon-General's Office.) Collated by H. L. THOMAS, M. D.

*Carotid.*

Case 1.—B. Creecy, company "E," Forty-Second Virginia regiment, wounded May 3, 1863, by a Minie ball, passing through the larynx above the vocal chords and carrying away the epiglottis. The common carotid of the left side was ligated on the 12th for excessive arterial hemorrhage. No chloroform could be administered. Patient fainted during the operation. On the morning of the 18th the right common carotid had to be ligated. Patient died thirty-eight hours after second operation. No brain symptoms supervened, and the heat of the head was retained. Autopsy: the hyoid artery of the left side was wounded; both carotids had been effectually secured.

Case 2.—J. W. Jones, company "E," Twenty-First Mississippi regiment; wounded May 3, 1863; ball entering left ear, fracturing the superior maxillary bones, and escaping at the right angle of the mouth. The wound began to slough on the 3d of June, and on the 5th, 6th and 7th hemorrhage occurred. Carotid artery was tied on the 7th, and patient died same day.

Case 3.—Richard Kelley, company "C," Sixth Louisiana regiment; wounded July 3, 1863. Division of external carotid by Minie ball. Common carotid ligated July 13, 1863. Left at Williamsport, Md.

Case 4.—Moses Hutts, aged 35. The lower jaw was badly shattered, and the tongue injured. The right common carotid was ligated June 7th; hemorrhage did not recur; patient died on the 8th.

Case 5.—J. H. McGuire, company "K," Twenty-Fourth Mississippi regiment; wounded September 26, 1863. Ball entered lower portion of the left mastoid process and lodged. Ligation of common carotid artery at upper third, Oct. 10th, 1863. Died on the 24th inst.

Case 6.—Daniel Shockley, company "I," One Hundred and First Indiana regiment; was wounded Sept. 20, 1863, by a round musket ball entering the face about an inch from the corner of the mouth, passing downwards and backwards, across the upper part of the neck, badly fracturing the lower jaw in its passage. The ball was extracted near transverse process of third cervical vertebra. The patient had hemorrhage from the entrance wound amounting to one quart on the 25th of September; hemorrhage recurred again on the 30th Three other hemorrhages, of October 6th, 9th and 10th, respectively, so reduced the strength of the patient, that the common carotid artery was ligated. The ligature separated on the 29th of October. Recovery perfect.

Case 7.—M. W. Smith; wounded May 5, 1864; ball entered the left temple just above zygoma, ranging downwards and backwards beneath the ear and immediately under the mastoid process, and passing out through the soft parts of the neck. On the 12th of May secondary hemorrhage supervened, which was controlled by pressure. It occurred from day to day until the 21st, when the common carotid was ligated. The hemorrhage was controlled, but the condition of the patient was anæmic, and he died in twenty-four hours, evidently from the want of supply of blood to the brain.

Case 8.—E. F. Lilley, company "G, Eighth Texas Cavalry, aged 24; was wounded May 9, 1864, by a Minie ball in the face. Secondary hemorrhage, to the extent of one pint, occurred on the 16th, apparently from wound in right side of the mouth. At six o'clock, P. M., there was a repetition of the hemorrhage, amounting to about three pints, from same point. The right primitive carotid was ligated in the inferior triangle, in the usual way, and with very little additional loss of blood. Chloroform was not used, and the patient bore the operation well. The operation was successful to the extent of arresting the hemorrhage, of which there was no recurrence. The patient died May 16, 1864, with well marked cerebral symptoms.

Case 9.—G. W. Nelson, company "K," Twelfth Georgia regiment; was wounded June 6, 1864, the ball entering posteriorly to left ear, passing upwards and forwards, and emerging at the infra-orbital ridge, fracturing the zygoma. The wound was extensive, and the hemorrhage considerable. The cavity was plugged after the removal of loose bone, but the hemorrhage returned the next day, and the external carotid was ligated. Hemorrhage recurred two days successively, in the last instance produced by patient's tearing off the dressings in his sleep. The patient died on the 19th.

*Subclavian.*

Case 1.—Corporal G. M. Caughman, company "K," Thirteenth South Carolina regiment, aged 25; wounded July 3, 1863, the ball passing through upper part of the chest, wounding the lung and the subclavian artery where it passes between the clavicle and first rib. The subclavian was ligated on the inner side of the clavicle. The operation was successful; the patient was furloughed, with the wounds entirely healed, but with the left arm paralyzed.

Case 2.—J. H. Kitrell, company "D," Third Tennessee regiment; wounded July 12, 1863, the ball fracturing humerus, and primary amputation being performed through surgical neck. The stump was progressing well until July 20, when slight hemorrhage occurred, which was controlled by pressure. Hemorrhage recurred again on the 28th, and digital pressure was diligently applied up to Aug. 2d, when the hemorrhage again took place more copiously than ever. Effort was then made to expose the bleeding vessel by tearing open the flaps, but the adhesions were too firm, and, the hemorrhage proceeding from two points, it was determined to ligate the subclavian at its third division. The patient, under chloroform, bore the operation well, but required stimulation, having lost largely of blood from the stump, but none from the operation itself. The separated flaps re-adhered, and the ligature came away on the fourth of September. The patient was furloughed the latter part of the month.

Case 3.—John T. Endy, company "F," Fifth North Carolina regiment, aged 23; wounded July 2, 1863, the ball entering one and a half inches below the scapula, ranging forwards, but having no exit. There was great tumefaction and effusion about the shoulder, while the wound under the deltoid region was filled with clots of blood. Hemorrhage supervened on the morning of the 16th, but was controlled by pressure and styptics; it occurred again the evening of the same day, and was controlled in like manner. On the morning of the 17th, very profuse hemorrhage took place, which could only be controlled by pressure over the subclavian artery. Exploring the wound failing to detect the bleeding vessel, it was determined to ligate the subclavian in its third division. The operation was performed without any untoward accident, but, while the hemorrhage was lessened, the flow of blood could not be entirely arrested in the wound, even with the assistance of styptics; it was therefore decided to ligate also the suprascapular artery, which had been exposed in the operation; this being done, the hemorrhage immediately ceased. The patient was put to bed with the arm warmly wadded, and at night there was sufficient temperature in the parts below the seat of ligature. The ligature from the suprascapular came away on the tenth day, and that from the subclavian on the thirteenth day. The patient got well without any bad symptom, and was furloughed on the 31st of August. The ball was not discovered.

Case 4.—W. S. Averitt, company "H," Fourteenth Tennessee regiment; wounded Aug. 9, 1862. Arm amputated just below surgical neck. Excessive hemorrhage having supervened, the subclavian was ligated at usual point. Died January 26, 1863.

Case 5.—A. C. Howard, aged 19; wounded May 31, 1862. Ball passed through left shoulder, injuring the spine and producing paralysis. June 7—ligation of subclavian artery in consequence of hemorrhage. Died June 18.

Case 6—J. W. King, company "C," Twenty-Ninth North Carolina regiment; wounded Sept. 19, 1863, the ball passing through shoulder joint, fracturing and detaching head of humerus. The ball entered near the coracoid process and passed out over the spine of the scapula. The accident was followed by a high degree of swelling and inflammation, extending from the seat of injury down the forearm; suppuration copious and offensive, with high irritative fever. On the 10th of October there was hemorrhage from the anterior wound, which was arrested by pressure; on the 11th, the hemorrhage recurred copiously from both wounds, and the subclavian was ligated in its external third. There was no further hemorrhage, but gangrene attacked the wound of operation on the 20th, and the patient died the next day.

Case 7.—Result fatal. (This case is reported in full in the February number of the Journal, by Surgeon Browne.)

We give below a tabular statement of the rest of the cases of ligation, including those detailed above:

| Vessel. | Cases. | Recovery. | Death. | Undetermined. |
|---|---|---|---|---|
| Carotid | 9 | 1 | 7 | 1 |
| Subclavian | 7 | 3 | 4 | 0 |
| Axillary | 7 | 2 | 5 | 0 |
| Brachial | 43 | 30 | 5 | 8 |
| Arteries of Forearm | 16 | 14 | 1 | 1 |
| Femoral | 53 | 24 | 24 | 5 |
| Profunda | 3 | 1 | 1 | 1 |
| Popliteal | 2 | 1 | 1 | 0 |
| Arteries of the Leg | 11 | 5 | 4 | 2 |
| All others, including Facial, Temporal and Occipital | 6 | 3 | 3 | 0 |
| Total | 157 | 84 | 55 | 18 |

In giving the above report, it is not pretended to include all the ligations performed, but simply all that have been reported. Medical officers can look over it and see to what an extent the deficiency lies at their own doors. That the bloody work through which the army has passed in four campaigns has *demanded* only 157 ligations, and many of these of only a trivial vessel, is an estimate which cannot be adopted. Certainly more operations of this character have been performed, for the number reported does not bear a respectable ratio in the chapter of gun-shot accidents. The presumption is, they have not been reported, and are sleeping in the *case-books* of the hospitals. Gentlemen may see to what a desirable use full reports of each special branch of surgery may be put—namely, the laying of them before the profession at large, and especially before those officers whose experience has not yet brought them in contact with such cases.

Even in many of the reports the data are so meagre as not to furnish any satisfactory conclusion with regard to the gravity of the case. Brevity is a very commendable feature in clinical reports, but should not be pushed to the extent of robbing the case of its interest. "Alexander died—Alexander was buried;" but there are some people who would be curious to know how he died and when he was buried; and it is a lean obituary that does not give these small items Some of the reports, indeed, seem to have been based upon the editorial warning that "funeral notices of more than —— lines will be charged for as advertisements." But we are satisfied there is room enough in the Surgeon-General's office for reasonable and instructive post-obits, and if they are transferred to the pages of this Journal, disquisitive platitude may be modestly retrenched, but deficient data cannot be supplied.

